# Gene Expression Is Not Random: Scaling, Long-Range Cross-Dependence, and Fractal Characteristics of Gene Regulatory Networks

**DOI:** 10.3389/fphys.2018.01446

**Published:** 2018-10-22

**Authors:** Mahboobeh Ghorbani, Edmond A. Jonckheere, Paul Bogdan

**Affiliations:** Electrical Engineering Department, University of Southern California, Los Angeles, CA, United States

**Keywords:** gene expression, gene regulatory network, fractals, dynamics, entropy

## Abstract

Gene expression is a vital process through which cells react to the environment and express functional behavior. Understanding the dynamics of gene expression could prove crucial in unraveling the physical complexities involved in this process. Specifically, understanding the coherent complex structure of transcriptional dynamics is the goal of numerous computational studies aiming to study and finally control cellular processes. Here, we report the scaling properties of gene expression time series in *Escherichia coli* and *Saccharomyces cerevisiae*. Unlike previous studies, which report the fractal and long-range dependency of DNA structure, we investigate the individual gene expression dynamics as well as the cross-dependency between them in the context of gene regulatory network. Our results demonstrate that the gene expression time series display fractal and long-range dependence characteristics. In addition, the dynamics between genes and linked transcription factors in gene regulatory networks are also fractal and long-range cross-correlated. The cross-correlation exponents in gene regulatory networks are not unique. The distribution of the cross-correlation exponents of gene regulatory networks for several types of cells can be interpreted as a measure of the complexity of their functional behavior.

## Introduction

Protein synthesis is fundamental for biological systems to perform a variety of functions. They control the organism’s shape or can function as enzymes catalyzing specific metabolic pathways to regulate specific cellular processes. These cellular functions include responding to stimuli, transporting molecules and catalyzing metabolic reactions. In order to program cells for performing the desired functionality, one should regulate the protein synthesizing process. The process of protein synthesis from the activation of a specific gene is called gene expression (Lockhart and Winzeler, 2000; Teichmann and Babu, 2004; Huang et al., 2005; Düvel et al., 2010).

**FIGURE 1:**
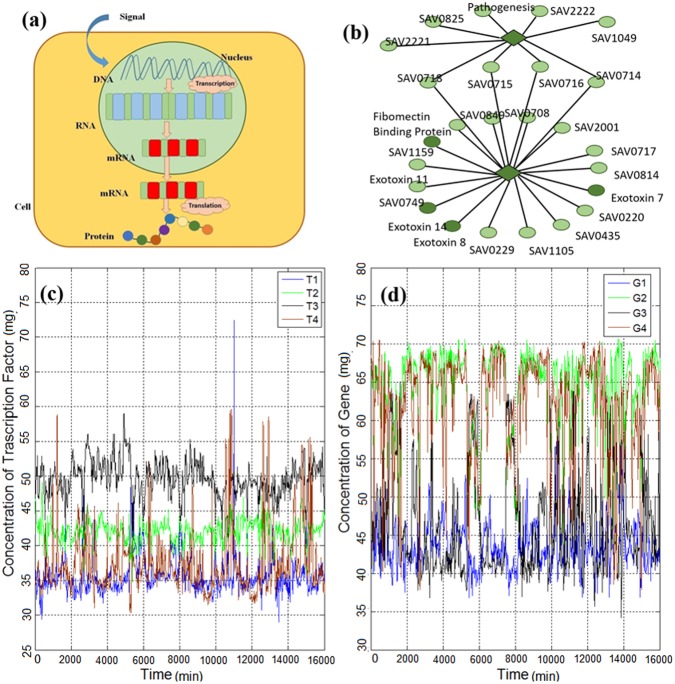
**(a)** Gene expression process is initiated with the triggering of a gene in the DNA strand and continues with the generation of RNA, mRNA and, finally, the protein product. **(b)** Part of the gene regulatory network of *Escherichia coli* reported by Lockhart and Winzeler (2000), which consists of genes and transcription factors (TFs). The diamonds represent TFs and the circles represent genes. **(c)** Time series of TFs of *E. coli* obtained from Marbach et al. (2012) and explained in the methods section. **(d)** Time series of genes of *E. coli* obtained from Marbach et al. (2012) and explained in the methods section.

Gene expression (briefly shown in Figure 1a) is the process in which the genetic information of a cell causes a cell to generate a functional gene product and, finally, perform specific cell functions (Niedenthal et al., 1996). In other words, it is the process by which genotype information gives rise to phenotype (observable characteristics). It is a vital process, which causes cellular differentiation, morphogenesis, and the versatility and adaptability of any organism (O’Connor et al., 2010). Controlling the production process of the desired gene expression product (e.g., a protein) refers

to the regulation of the gene expression process. The regulation of gene expression controls the amount and timing of production of target proteins (Malone et al., 2009). Hence, investigating the dynamics of gene expression enables to understand the mechanisms driving biological organisms. This knowledge helps us from both scientific and engineering perspectives. It can be exploited to detect an anomaly or disease or to engineer cells for performing specific tasks (e.g., drug delivery for cancer treatment) as it is the target of synthetic biology.

The biophysical mechanism affecting the regulation process has been actively studied (Elf et al., 2007; Kolesov et al., 2007; Kuhlman and Cox, 2012; Bauer and Metzler, 2013; Pulkkinen and Metzler, 2013). For instance, searching for the target gene by the transcription factors (TFs) is discussed in Kolesov et al. (2007); Pulkkinen and Metzler (2013) and the diffusion process of search for the target genes is studied in Elf et al. (2007); Bauer and Metzler (2013). Also, the spatial distribution of gene products is reported in Kuhlman and Cox (2012). However, these prior studies were not concerned with the mathematical characterization of the gene expression dynamics for several gene regulations in a network of genes. To identify the main mathematical characteristics of gene expression dynamics, we investigate individual and cross-dependent gene expression time series. First, we investigate the statistical properties of single (isolated) gene expression time series (shown in Figures 1c,d), and, then, we analyze the cross-correlation between pairs consisting of a gene and a TF in the gene regulatory network (Figure 1b). In contrast to the previous study (Tsuchiya et al., 2016) in which regulation of cell fate through genome-wide expression by temporal-spatial self-organization is considered, here, we mainly analyze the temporal variability of individual genes. We then investigate the correlation of linked TF and genes. Moreover, we analyze the expression level of single cells without considering population effect (Tsuchyia et al., 2007).

The remaining of this paper is organized as follows: In the first part of the Results section, we present our findings on the individual and cross-dependence dynamics of gene expression time series. Then, we report the distribution of the cross-dependencies and a complexity quantification strategy for the gene expression networks. In the latest section of Results, we investigate whether the observed multifractality can be explained by a well-known model such as the Mandelbrot analytical cascade model. The Discussion section concludes our findings and outlines some future research directions. Lastly, the Methods section summarizes the mathematical strategies used for obtaining the findings reported here.

## Results

### Gene Expression Dynamics Exhibits Long-Range Dependency and Multifractal Properties

**FIGURE 2:**
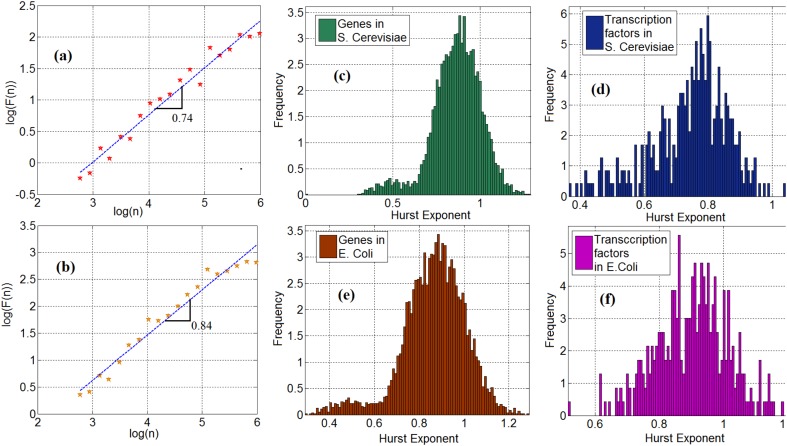
**(a)** Scaling of fluctuation function of a gene time series in *Saccharomyces cerevisiae*, **(b)** Scaling of fluctuation function of ynel gene time series in *E. coli*, **(c)** Histogram of cross-correlation exponent of genes in *S. cerevisiae*, **(d)** Histogram of cross-correlation exponent of TFs in *S. cerevisiae*, **(e)** Histogram of cross-correlation exponent of genes in *E. coli*. **(f)** Histogram of cross-correlation exponent of TFs in *E. coli*.

We investigate the statistical properties of gene expression data and compute the Hurst exponents of gene expression time series in *Saccharomyces cerevisiae* (*S. cerevisiae*) and *Escherichia coli* (*E. coli*). Figures 2a,b show the log–log plot of the fluctuation function as a function of the scale for the time series of a TF (ynel) in *S. cerevisiae* and *E. coli*, respectively. In these plots, the slope of the curve represents the Hurst exponent. We observe that 95 and 98% of the time series of genes from the *S. cerevisiae* and the *E. coli* gene expression networks, respectively, exhibit a long-range dependency property. More precisely, their Hurst exponent was greater than 0.5. To demonstrate this important property, Figures 2c,e show the histogram of the Hurst exponent of gene expression time in *S. cerevisiae* and *E. coli*, respectively. Generally speaking, a Hurst exponent that exceeds the 0.5 threshold value denotes a persistent (positively correlated) behavior in the sense that a high value is likely to be followed by another high value with nonzero probability. In addition, because the Hurst exponent for most of the genes is significantly higher than 0.5, the gene and TF time series cannot be regarded as a random process and modeled through Markovian formalism (Kantelhardt et al., 2001). This mathematical characteristic provides a clue as to how to construct stochastic models for gene expression processes, but this is left for future work. We observe the same property in the time series of TFs in *S. cerevisiae and E. coli*. More precisely, 97% of the TFs in *S. cerevisiae and E. coli* possess the long-range dependence property. The histogram of the Hurst exponent of TFs in *S. cerevisiae* and *E. coli* are shown in Figures 2d,f respectively.

Employing fractal analysis is also helpful to gain insight into other interesting properties. Here, we see a bimodal characteristic in the Hurst exponent distribution, shown in Figures 2c–f. This feature is especially visible in Figures 2c,e where the histogram of genes in *S. cerevisiae* and *E. coli* is presented. This may be explained by a possible bimodal diffusion potential, as in Muzychuk (2006). Since gene expression includes a diffusion process with multiple diffusion potentials (inside and outside the nucleus), this bimodality can be explained by non-equilibrium Brownian motion with multiple potential profiles. However, further experimental studies are required to elucidate the nature and implications of these bimodal statistics.

**FIGURE 3:**
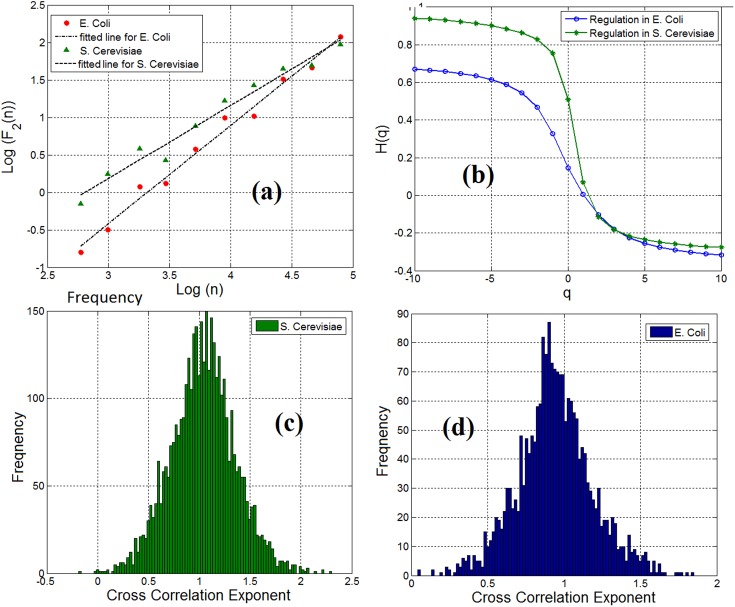
**(a)** Scaling of detrended covariance function in a gene-TF link in *E. coli* (ihfB to ompR) and *S. cerevisiae* (YLR256W to YKL020C) regulatory network. **(b)** Generalized cross-correlation exponent of a gene-TF link in *E. coli* and *S. cerevisiae*. **(c)** Histogram of cross-correlation exponents of gene regulatory network links in *S. cerevisiae* and **(d)** Histogram of cross-correlation exponents of gene regulatory network links.

By employing the multifractal detrended fluctuation analysis (MFDFA) (Kantelhardt et al., 2002) (see Materials and methods section) to examine the multifractal characteristics of gene expression time series, we observe that both genes and TFs have pronounced multifractal properties. For monofractal behavior, the generalized Hurst exponent displays a linear dependency with the order *q* of the cross moments. Instead, if the generalized Hurst exponent exhibits a nonlinear dependency (such as the S-shape displayed in Figure 3b) as a function of the order *q* of the cross-moments, then the stochastic interdependence is considered to possess multifractal characteristics.

To provide a more in-depth report, we use the bootstrapping technique (Efron, 1982) to investigate the existence of the long-range dependence property, considering the limitations related to the length of the experimental time series. For every gene expression time series, we have sampled 10 randomized subintervals of the gene expression time series, each containing 90% of the ordered piece of the original time series. Then, we calculate the Hurst exponents for all the randomized versions. The difference between the percentage of the long-range dependency for the gene expression time series and the randomized versions was approximately 0.006 for *E. coli* and 0.0001 *S. cerevisiae*. We also investigate whether the observed Hurst exponent varies in different conditions. We observed that for time series of *E. coli*, the Hurst exponent varies in different acidic levels and osmotic stress level and we have reported them in Supplementary Material.

### Time Series of Interactions Within the Gene Regulatory Networks Demonstrate Long-Range Cross-Correlation and Multifractal Properties

We analyzed the cross-correlation between linked pairs of genes and TFs in gene regulatory networks. By computing the cross-correlation exponent (Podobnik and Stanley, 2008), we noticed that 98% of the linked pairs of genes and TFs in gene regulatory network for *E. coli* and *S. cerevisiae* possess the long-range dependence property. Figure 3a shows the scaling of the detrended covariance function for a pair of gene and TF (link) in *E. coli* (ihfB to ompR) and *S. cerevisiae* (YLR256W to YKL020C). We have applied the multifractal detrended cross-correlation analysis for pairs of genes and TFs (links) in the gene regulatory network of *E. coli* and *S. cerevisiae* and found that there is a pronounced multifractal cross-correlation signature in these gene regulatory network links. Figure 3b shows the generalized Hurst exponent *H*(*q*) as a function of the order of the cross moments *q* in Figure 3a. For a mono-fractal behavior, the generalized Hurst exponent displays a linear dependency with the order *q* of the cross-moments. Instead, if the generalized Hurst exponent exhibits a nonlinear dependency (such as the S-shape displayed in Figure 3b) on the order *q* of the cross-moments, then the stochastic interdependence is considered to possess multifractal characteristics. In conclusion, the causal relationship between TFs and genes in gene regulatory networks was mainly also long-range dependent. The concentration level of a gene not only depends on the current concentration level of the linked TF but also on the previous values of that gene. This dependency obeys a power-law-like relationship.

### The Distribution of Cross-Correlation Exponents of Pairs of Genes and Transcription Factors of Gene Regulatory Networks Has a Wide Range of Variation

Although we observe the fractal and long-range cross-correlation in linked pairs of genes and TFs in the gene regulatory networks, the cross-correlation exponents were not the same in all the links. We have shown the distribution of the cross-correlation exponents for pairs of genes and TFs in the *S. cerevisiae* and *E. coli* gene regulatory networks in Figures 3c,d respectively. Inspired by Shannon entropy (Shannon, 2001), we use this histogram for measuring the entropy, and hence, the information content of a gene regulatory network across different cell types for quantitative analysis and specification of gene regulatory networks. The computed Shannon sample entropy for *S. cerevisiae* and *E. coli* was 4.18 and 5.29, respectively. Consequently, we conclude that the gene expression network of *E. coli* has more complex dynamics than that of *S. cerevisiae*. Also, considering a static gene regulatory network and having traces of gene expression time series for a cell at different times, we can compute the cross-correlation exponents for the links at a different time. This can be useful to compare statistical properties and complexity of dynamics. Similarly, by having different time series of gene expression dynamics, we can compare normal vs. disease affected (for example cancer typed) cells.

### Multifractal Characteristics of Interactions Within the Gene Regulatory Network Can Be Modeled by Random Cascades on Wavelet Dyadic Trees

We analyzed the multifractal property of the cross-correlation of pairs of genes and TFs in a gene regulatory network. We investigated whether the observed multifractality can be explained by the known analytical cascade models including the Mandelbrot bimodal cascade model (Mandelbrot et al., 1997) (see Materials and methods section) and the random cascades on wavelet dyadic trees (Arneodo et al., 1998). We observe deviations of the empirical spectrums from the Mandelbrot model and an approximate agreement to the random cascades on wavelet dyadic trees model.

**FIGURE 4:**
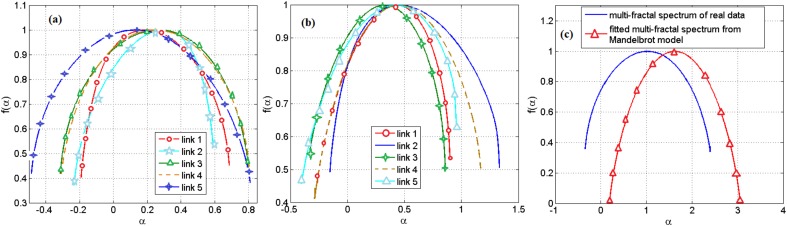
**(a)** Multifractal spectrum of several randomly picked links of gene regulatory network of *E. coli*. **(b)** The multifractal spectrum of several randomly picked links of gene regulatory network of *S. cerevisiae*. **(c)** The multifractal spectrum of a link between the gene regulatory network of *S. cerevisiae* and the best spectrum from the Mandelbrot binomial model.

Based on the range of the Holder exponent values in the multifractal spectrum, we observe that only 0.04 of the links in a gene regulatory network of *S. cerevisiae* and none of the links in the network of *E. coli* can be modeled by the Mandelbrot cascade model for multifractal spectrums (see Materials and methods section). We observe that even for the few links that we could find a closest Mandelbrot model spectrum, the deviation from the Mandelbrot model and the data we had for gene regulatory network was significant. We show two such samples in Figure 4. Figure 4a shows several multifractal spectrums for the links in *E. coli* gene networks. Note that the peak of the multifractal spectrum for these spectrums was lower than the value 1, which does not fit with the Mandelbrot Binomial Cascade Model (Mandelbrot et al., 1997). Figure 4b shows several multifractal spectrums for the links in *S. cerevisiae*. Figure 4c shows the closest Mandelbrot Model we could fit for the links in the *S. cerevisiae* gene regulatory model. There is a significant deviation between the Mandelbrot model and the spectrum from gene regulatory network data.

**FIGURE 5:**
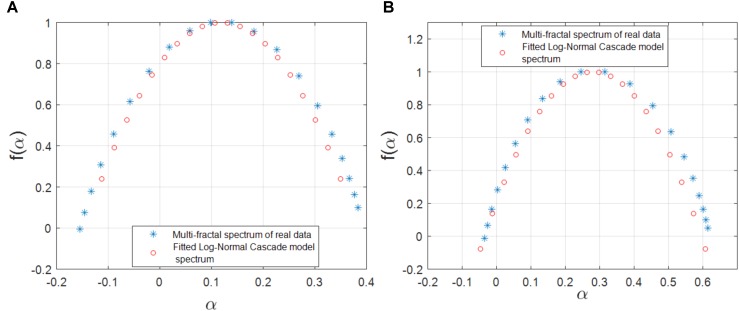
**(A)** The multifractal spectrum of a cross-dependency (link) between the gene regulatory network of *E. coli* (soxR and soxS genes) that can best be fitted by the log-normal cascade multifractal model. **(B)** The multifractal spectrum of a cross-dependency (link) between the gene regulatory network of *S. cerevisiae* (YKL032C and YKL043W genes) that can best be fitted by the log-normal cascade multifractal model.

We also investigated the agreement between the observed multifractality of the cross-dependencies in the gene regulatory network of S. cerevisiae and *E. coli*, respectively, and a few well-known multifractal models such as the random cascades on wavelet dyadic trees (Arneodo et al., 1998). We investigated whether the log-normal W-cascade model can be fitted to the cross-dependencies (links) in the two above-mentioned gene regulatory networks. We extracted the parameter of the estimated log-normal W-cascade model based on the peak of the empirical spectrum and the variation of the singularity spectrum (see the Methods section). We observed very similar spectrums for a significant number of links. We computed the overlapping area under the curve for both the estimated and the empirical multifractal spectrums. The ratio of the area of the overlapping fitted spectrum to the area of the empirical spectrum can be used to either accept or reject the postulated multifractal model as a good fitting for the empirically estimated multifractal spectrums. In this study, we used two threshold values of 70 and 75% for the ratio of mentioned areas. For the gene expression cross-dependencies in *E. coli*, we observed a 74 and 38% agreement between the postulated model and the empirically estimated multifractal spectrums when considering overlapping area ratio thresholds of 70 and 75, respectively. For the gene expression cross-dependencies in *S. cerevisiae*, we observed a 59 and 31% agreement between the postulated multifractal model and the empirically estimated multifractal spectrums when considering overlapping area thresholds of 70 and 75%, respectively. Figures 5A,B show a best fitting scenario between the postulated multifractal model and an empirically estimated spectrum for a cross-dependence in the gene regulatory network of *E. coli* and *S. cerevisiae*, respectively.

**FIGURE 6:**
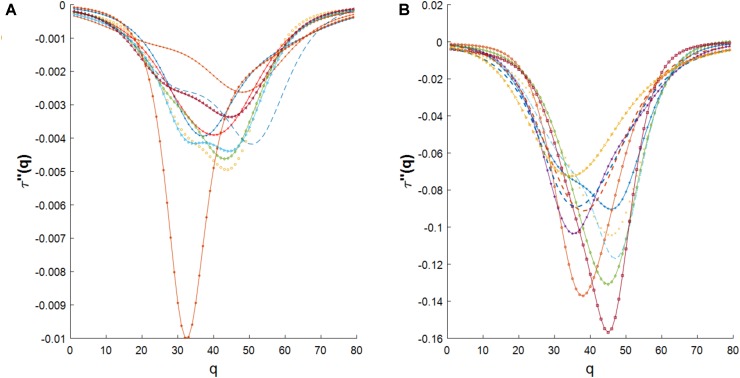
**(A)** The second derivative of the mass exponent for cross-dependencies in *E. coli.* **(B)** The second derivative of the mass exponent for cross-dependencies in *S. cerevisiae*.

We also investigated the agreement between the empirical multifractal spectrums and the log-Poisson W-cascade model (Arneodo et al., 1998). We observed that the empirical multifractal spectrums could not be described by this cascade model since the second derivative of the mass exponent should follow a power law (see Methods section) while the empirical data has a significant deviation from a power law trend (see Figure 6 in the Methods section).

## Discussion

A genome expression vector is the most informative descriptor of a cell state, as the functional state of an organism is determined largely by the pattern of expression of its genes (O’Connor et al., 2010). Gene expression is the process in which information from a gene is used to synthesize a functional gene product. It is the process in which the information flows within a complex biological system. As the search for patterns in nature and their interpretation is one of the main purposes of science, unveiling the DNA patterns in those sequences has become an exciting challenge to the present generation of biologists, statistical physicists, and information scientists. Toward this end, many researchers have studied the statistical properties of coding and non-coding segments of DNA sequences. They have reported interesting results showing fractal nature of coding DNA regions (Lockhart and Winzeler, 2000; Teichmann and Babu, 2004; Huang et al., 2005; Düvel et al., 2010). However, these studies fail to address the dynamical properties of the biological systems. Since biological systems are dynamic, their study requires monitoring their activity at multiple time points.

To investigate the causal relations in gene expression, numerous biophysical mechanisms affecting the regulation process were studied in Elf et al. (2007), Kolesov et al. (2007), Kuhlman and Cox (2012), Bauer and Metzler (2013), Pulkkinen and Metzler (2013). It is demonstrated by several simulations that rapid and reliable gene regulation requires that the TF be close to their target site on DNA (Kolesov et al., 2007). In Pulkkinen and Metzler (2013), the authors use an explicit model for numerical analysis. The authors report that the observed variations in regulation efficiency are linked to the magnitude of the variation of TF concentration peaks as a function of binding site distance from the source. In Bauer and Metzler (2013), the authors have presented a semi-analytical model for the *in vivo* target search of the TFs within a diffusion framework. They have shown that alternating between three-dimensional bulk diffusion and one-dimensional sliding along the DNA contour can provide a quantitative approach to gene regulation in living bacteria cells. Their proposed model agrees with experimental findings regarding the mean search time of lac repressor in a living *E. coli*. In Elf et al. (2007), the authors have reported their observation of kinetics of the gene expression process at the single-molecule level in living cells by labeling with a fluorescent protein, which agrees with 1D diffusion along DNA segments and 3D diffusion. In Kuhlman and Cox (2012), the authors test the expectation of previous theoretical models using high-throughput single-molecule microscopy to determine the average spatial distribution of lac repressor. Their finding shows inconsistency between expectations and experimental findings. They show that the gene products distribution is spatially inhomogeneous and dependent on the location of the repressor gene in bacteria and eukaryotes. However, they do not consider the gene expression dynamics from a network of genes perspective and do not account for cross-correlations and multiscale phenomena.

To study the dynamic nature of gene expression processes, researchers must monitor activity levels of genes and TFs at multiple time points. The most informative source of information regarding gene expression activity is the gene expression time-series. With advances in gene array technology, the level of gene expression of thousands of genes (by providing the concentration level of gene expression products) can be measured simultaneously. By accessing a high- throughput data collection, a wide range of insights, such as characterizing the functions of specific genes, the relationships among these genes, and their regulation and coordination can be gained. These insights can also be used to understand the gene regulatory network as a complex network. There are many studies which try to infer the underlying gene regulatory network from empirical time series (Marbach et al., 2012). However, little is known about the mathematical characteristics of the gene expression dynamics from a complex systems perspective.

In this paper, we investigated the scaling properties of gene expression dynamics. Unlike previous work that demonstrated the fractal properties in DNA sequences (Peng et al., 1994; Arneodo et al., 1996; Zhang et al., 2011; Marbach et al., 2012), we investigate the dynamics of cross-dependencies between genes and TFs within the gene regulatory networks. We show that the gene expression time series (which is the concentration of gene expression products in the process of gene expression) have fractal and long-range dependence properties in *E. coli* and *S. cerevisiae*. We also investigate the cross-correlation of gene-TFs, which are linked together in gene expression networks. We report the fractal and long-range cross-dependency of linked genes and TFs of gene expression networks in *E. coli* and *S. cerevisiae*. We also show that the multifractal nature of these cross-correlations cannot be modeled through a Mandelbrot binomial cascade model. In contrast, we found very good agreement between the empirical multifractal spectrum of the cross-dependencies in the gene regulatory networks and the log-normal *W*-cascade model. We suggest investigating more cascade models on empirical data (Bacry and Muzy, 2003; Chainais et al., 2005; Kiyono et al., 2007) as future work. In summary, there is a need for more advanced theoretical models that can capture the multifractality observed in this critical biological process. One possible method for modeling gene expression dynamics can exploit the multifractal Fokker-Planck formalism, as discussed in Xue and Bogdan (2017).

We also propose using the distribution of cross-correlation exponent of the links in gene regulatory network as a measure of the complexity for the regulatory networks. Having this complexity measure enables a quantitative descriptor for different cell types or to differentiate different cell fates when the system undergoes transitions. We report the distribution of cross-correlation exponent of links in regulatory networks of *E. coli* and *S. cerevisiae* as case studies. We suggest investigating this property on a wider range of biological systems when enough data sets are available. We also propose using this property as a network property in general. We propose using the distribution of cross-correlation exponents of gene-TF links in a complex network to measure the complexity of the interactions in the network. Also, the computed cross-correlation exponents of a network can be used by other algorithms, such as those proposed in Anand and Bianconi (2009), Anand et al. (2011), Teschendorff and Enver (2017) for computing the entropy of a network. This has a variety of applications in distinguishing the different status of cells (e.g., healthy vs. disease affected states). This can reveal insightful results in many complex networks either in biology, social, financial and many other interesting examples of complex networks.

Our findings explain the inherent variability in gene expression processes, even among isogenic cells situated in an identical environment. Because of the long-range cross-dependency of a gene and its linked TF, the current concentration level of a gene depends both on current and previous values of its own and its linked TF. As explained in previous studies, this leads to phenotypic diversity, which can be helpful for surviving in an uncertain and fluctuating environment (Ji et al., 2013). Also, the endogenous cellular mechanism through positive and negative feedback controls variability in gene expression to prevent disruption from normal development. Hence, unlike the usual assumption about noise as a nuisance, variability in gene expression makes the population of cells more robust against environmental fluctuations. Interestingly, there are other examples in nature in which the presence of noise makes the system smarter. For example, in Maass (2014), they have shown how the presence of noise in a network of spiking neurons in the human brain enables probabilistic reasoning and creative problem-solving.

This study is the first to demonstrate the long-range dependency of gene expression dynamics. In contrast to previous studies (Bernaola-Galván et al., 1996), which have shown the long-range dependency for the structure of DNA, we investigate the dynamics of gene expression time series. Previous studies show that coding regions of DNA structure, which store the biological information for the gene expression process, possess the long-range dependency property (Bernaola-Galván et al., 1996). In contrast, our results report the same property in gene expression time series. Of note, these dynamics stem from the transformation of information from the structure to dynamics by producing gene products. This is an insightful empirical result that can trigger more studies on other examples from nature, as well as analytical and mathematical investigations. For example, investigating other processes that follow a rule from a static structure to generate dynamical products and process (such as the central dogma of biology) can be interesting. Lastly, mathematical and analytical investigation of the relation between structure and dynamics of processes are also fundamental in theory. It would be revealing to investigate how long-range dependency (and/or fractal/multifractal properties) evolves from structure to dynamics (and vice versa) in processes. Answering to the question of how long-range dependency transfers between structure and dynamics and how the degree of fractality/multifractality of structure and dynamics are like each other would have a huge impact on predicting the behavior of complex systems.

## Materials and Methods

We use the data set from the publicly available DREAM project^1^ (Stolovitsky and Califano, 2007), which is for assessment of network inference methods. It is organized around annual challenges where the community of network inference experts is solicited to run their algorithms on benchmark data sets. The data is provided from Gene-expression microarray datasets for *E. coli* and *S. cerevisiae*^2^. A compendium of microarray data is compiled for *E. coli*, where all chips are on the same Affymetrix platform, the *E. coli* Antisense Genome Array. In total, 805 chips with available raw data Affymetrix files were compiled. Completion of microarray normalization and filtering resulted in a total of 4,297 genes over the 805 microarrays. Also, a compendium of microarray data was compiled for *S. cerevisiae*, where all chips are on the same Affymetrix platform, the Affymetrix Yeast Genome S98 Array. Chips were downloaded from GEO (Platform ID: GPL). In total, 536 chips with available raw data Affymetrix files were compiled. The completion of microarray normalization and filtering resulted in a total of 5,667 genes over the 536 microarrays. Transcriptional interactions and, hence, gene regulatory networks for *E. coli* and *S. cerevisiae* are collected from strong experimental supports in Marbach et al. (2012). Known transcriptional interactions for *E. coli* are collected from manually curated Ecocyc (Gama-Castro et al., 2010) and RegulonDB (Keseler et al., 2010) databases. A gene regulatory network for *E. coli* is constructed from RegulonDB Release 6.8. Only transcriptional interactions with at least one strong piece of evidence were included (2,066 interactions). For *S. cerevisiae*, we use the network based on the most stringent thresholds from MacIsaac et al. in MacIsaac et al. (2006) compared to other studies (Hu et al., 2007; Abdulrehman et al., 2010). By varying the thresholds required for binding and evolutionary conservation of motifs, different versions of the network were obtained. Based on the most stringent thresholds, which includes only interactions with strong evidence of binding and a strongly conserved motif, the interactions in the regulatory network are obtained. There are 5950 time-series, each having 536 data points for *S. cerevisiae*, and 4511 time-series, each having 805 data points for *E. coli* expression series.

### Noise in Gene Expression Time Series

Since DNA, RNA, and proteins involved in the gene expression process can be present and active at a few copies per cell, this process is sensitive to stochastic fluctuations (Raser and O’shea, 2005). The four most important sources of variation in gene expression dynamics include (i) the inherent stochasticity of biochemical processes that are dependent on the small number of molecules, (ii) differences in the internal states of cells, (iii) subtle environmental differences, and (iv) genetic mutations (Raser and O’shea, 2005). The existence of this variation causes genetically identical organisms with identical environmental exposure to varying in behavior and shape. The fluctuation in the gene expression process is inevitable and does not follow the law of mass action. Hence, in this study, we have investigated the process independently of assuming stationarity and the MFDFA method explained below is used.

### The Hurst Exponent and Multifractal Detrended Fluctuation Analysis (MFDFA)

In this paper, we have used the MFDFA method for analysis of gene expression time series. This method, which is the extension of detrended fluctuation analysis (DFA) to extract the Hurst exponent (Kantelhardt et al., 2002), is introduced in Kantelhardt et al. (2002) for analysis of multifractal properties of nonstationary time series. Since we do not have the stationarity assumption for gene expression time series, MFDFA method is a suitable one for studying them. Scaling properties and long-range dependency of time series can be obtained by the DFA method. However, for time series with multifractal properties and different scaling exponents on different scaling regimes or different time intervals, it is essential to exploit the multifractal detrended fluctuation method (MFDFA) to reveal the multifractal property.

The MFDFA method consists of five steps to estimate the multifractal spectrum of a nonstationary time series. Similar to the DFA method, the *profile* of the time series is obtained first, which is determined by the integration of the difference of the time series with its average value ($\hat{x}$):

$$y(i)=\sum_{\text{i}=1}^{k}\left(x(i)-\hat{x}\right)$$

Second, it divides the profile into non-overlapping segments (or scales (*n* = N/s), where *s* is the scale). For each of these boxes, a least squared local trend is fitted.

Third, it calculates the local trend within each segment. For each of these boxes, a least squared local trend is fitted. The value of the fitted time series obtained for boxes of length (*n*) is denoted by y_n_.

Fourth, it computes the average of the fluctuation function over all segments to obtain the *q*th order fluctuation function.

$$F{\left(s\right)}_{q}=\sum_{\text{k}=1}^{Ns}{\left({\left(y(i)-y_{n}(i)\right)}^{2}\right)}^{q}/Ns$$

Finally, the Generalized Hurst exponent is estimated by fitting a linear line to the log-log plot of the *F*(*s*)_q_ with respect to scale (*s*), according to the following equation:

$$F{\left(s\right)}_{q}=s^{H(q)}$$

The Hurst exponent is the value of the Generalized Hurst exponent (H(*q = 2*)), which is a special case and is used usually when one is interested only in analyzing the long-range dependency of a signal and not the multifractal chrematistics.

Finally, the multifractal spectrum of the multi-variable signal (α,f(α)) is estimated by the Legendre transform:

$$\begin{array}{c} \tau (q)=H(q)*(q-1)\\ \alpha (q)=d\tau (q){/}_{dq}\\ f(\alpha )=q\alpha -\tau (q) \end{array}$$

### Detrended Cross-Correlation Analysis (DCCA)

This method is designed to investigate the power law cross-correlation between two time-series (Podobnik and Stanley, 2008). Similar to the DFA (discussed in the previous sub-section), which computes the scaling behavior of the auto-correlation function, the DCCA method computes the scaling behavior of the cross-correlation function between two time-series and analyzes its scaling behavior.

DCCA method first computes the integrated *profile* of each time series:

$$\begin{array}{c} y_{1}(i)=\sum_{\text{i}=1}^{k}\left(x_{1}(i)-\overset{⌢}{x_{1}}\right)\\ y_{2}(i)=\sum_{\text{i}=1}^{k}\left(x_{2}(i)-\overset{⌢}{x_{2}}\right) \end{array}$$

Second, both the entire time series is divided into non-overlapping intervals. Third, it computes the local trend in each interval for each time series (*y*_1,*n*_(*i*), *and y*_2,*n*_(*i*)). Fourth, it calculates the covariance of the residual of profiles from local trends. It calculates the detrended covariance (H(q)) by summing over all segments of the nonstationary time series:

$$\hat{F}{\left(s\right)}_{q}=\sum_{\text{k}=1}^{Ns}({\left(y_{1}(i)-y_{1,n}{\left(i\right)}^{2}{\left(y_{2}(i)-y_{2,n}(i)\right)}^{2}\right)}^{q}/Ns$$

The cross-correlation exponent (λ) is estimated by fitting a linear line to the log-log plot of the $\hat{F}$(*s*)_q_ with respect to scale (*s*):

$$\hat{F}(s)q=s^{\lambda }$$

### The Mandelbrot Binomial Cascade Model

This model is proposed by Mandelbrot et al. (1997) to better explain an alternative for probability distribution for the erratic or fractal appearance of a probability measure. It starts with a probability measure (μ), which is self-similar:

μ([a,b]) = _m_0__ μ_([2a,2b])_ + _m_1__ μ_([2a-1,2b-1])_

Once the unit interval [0,1] is divided into two subintervals, _m_0__ mass is assigned to the left subinterval and _m_1_=1-m_0__ is assigned to the right subinterval. Repeating this step for each of the subintervals for *n* times will result in the Mandelbrot model with *n* iterations. Mandelbrot has proved that the limit behavior of this model when n is infinitely large (∞) can be best illustrated by multifractal formalism. He formulated the (α, f(α)) spectrum on the basis of the parameters of the Mandelbrot Cascade model. We have compared the observed multifractality spectrum in gene expression time series to the closest one obtained by Mandelbrot cascade model.

### Random Cascades on Wavelet Dyadic Trees

This model (Arneodo et al., 1998) is proposed to model multifractal objects. The notion of cascade here refers to a self-similar process whose properties are defined multiplicatively in different scales. In summary, in this model, the wavelet coefficients of a function are self-similar at different scales. Two types of W-cascades are proposed: Log-Normal *W*-cascades and Log-Poisson *W*-cascades. For the log-normal cascade (with μ and σ for parameters of the normal random variable), the following equation holds for singularity spectrum:

$$F(\alpha )=-\frac{\ln2}{2\sigma^{2}}{\left(\alpha +\mu /\ln2\right)}^{2}$$

For the Log-Poisson *W*-cascades, the following equation holds for the mass exponent:

$$\tau (\text{q}){=}_{\frac{1}{\ln2}(\lambda (1-\delta \text{q)-}\gamma \text{q)}}-1$$

As can be seen, the second derivative of the mass exponent for Log-Poisson *W*-cascade model has the following equation:

τ′(q)=λln2(−lnδδq)−γln2τ″(q)=−λln2(lnδ)2δq

We have reported the similarity of the multifractal spectrum of cross-dependencies in gene expression time series to the log-normal *W*-cascades model. Also, we have reported the disagreement of the multifractal spectrum of cross-dependencies in gene expression time series to log-Poisson *W*-cascade model due to its deviation from power-law shape as shown in Figure 6.

### Entropy and Entropy of a Network

Shannon entropy (Shannon, 2001) is a measure of *the unpredictability* of the state, or equivalently, of its *average information content*. Shannon defined the entropy of a discrete random variable X with possible values of {_x_1__,_x_2__,...,_x_k__} and probability mass function P(X) as:

H(X)=E[I(X)]=E[−logP(X)]

More explicitly, entropy can be written as:

$$H(X)=-\sum_{\text{i}=1}^{n}P(x_{\text{i}})\log P(x_{\text{i}})$$

Entropy is a measure of the unpredictability of the state or its average containing information. One example to illustrate is when there is no uncertainty and the random variables take only one value in which the value of the entropy will be zero. As the number of possibilities increases, the entropy increases as well.

**FIGURE 7:**
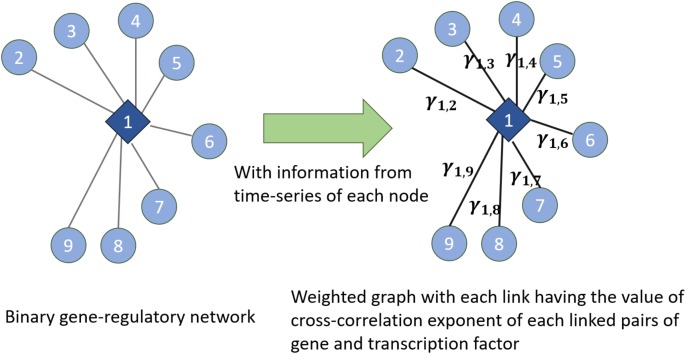
Extracting a weighted graph from cross-correlation exponents of the linked nodes in a gene regulatory network

We have used the notion of entropy in the context of networks. We consider the weight of the links in the network as the random variable and we discuss entropy of the weight of the weighted links. Given an undirected binary graph of gene regulatory networks and time series of genes and TFs in the network (which are the nodes in the gene regulatory network), we construct a weighted network (shown in Figure 7). In the constructed weighted network, the weight of each link is the *cross-correlation exponent* of the time series of two time-series linked together in the gene regulatory network. Then, in the new constructed weighted network, we consider the distribution of the weights of the links and entropy of them as a measure of the entropy of the network. Also, this weighted network can be used for other algorithms measuring the entropy of complex networks proposed in Anand and Bianconi (2009), Anand et al. (2011). Figure 7 illustrates this method by showing how the weights are assigned

to each link. In this figure, γ_1,2_,…, γ_1,9_ are the cross-corrections of the time series of the TF and genes which are linked together in the gene regulatory network in the left part of the figure. Hence, this shows how knowing the existing interactions in the network and having the time series of each node’s dynamics can lead us to know cross-correlation exponents and then assigning the concept of entropy to the network dynamics.

## Author Contributions

All authors listed have made a substantial, direct and intellectual contribution to the work, and approved it for publication. PB conceived the idea. MG, EJ, and PB discussed the research problem and approaches, identified analytical challenges and solutions. MG implemented the research discussions and performed the research.

## Conflict of Interest Statement

The authors declare that the research was conducted in the absence of any commercial or financial relationships that could be construed as a potential conflict of interest.

## Footnotes

1 http://wiki.c2b2.columbia.edu/dream

2 https://www.synapse.org/#!Synapse:syn2787209/wiki/70349
