# Supplementary material for: Gene Expression Is Not Random: Scaling, Long-Range Cross-Dependence, and Fractal Characteristics of Gene Regulatory Networks
Source: Front Physiol. 2018 Oct 22;9:1446. doi: 10.3389/fphys.2018.01446 (PMC6232942; doi:10.3389/fphys.2018.01446)
Supplement: Supplementary file 1 [file Presentation_1.PDF]

Comparing the scaling behavior of transcriptome of ompR null strains with wild-type strain of E. coli during acid or osmotic stress

To compare the scaling behavior of gene expression time series of E. Coli in different conditions (acidity level and responds to osmotic stress level), here we report the Hurst exponent of the time series of transcriptome of ompR null strains with wild-type strain of E. coli which we gathered from database in [1]. As can be seen the scaling behavior changes in different conditions.

Condition	Description	Hurst Exponent
E.coli_WT pH 5.6 A	genotype: Wildtype<br>E. coli strains was grown in MgM media at pH 5.6, 7.2 and 7.2 with 15% (w/v) sucrose to O.D ~0.6.	0.815
E.coli_WT pH 5.6 B	genotype: Wildtype<br>E. coli strains was grown in MgM media at pH 5.6, 7.2 and 7.2 with 15% (w/v) sucrose to O.D ~0.6.	0.708
E.coli_ΔompR pH 5.6 A	genotype: delta_ompR<br>E. coli strains was grown in MgM media at pH 5.6, 7.2 and 7.2 with 15% (w/v) sucrose to O.D ~0.6.	0.873
E.coli_ΔompR pH 5.6 B	genotype: delta_ompR<br>E. coli strains was grown in MgM media at pH 5.6, 7.2 and 7.2 with 15% (w/v) sucrose to O.D ~0.6.	0.913

[1]. https://www.ncbi.nlm.nih.gov/geo/
